# BRAF Signaling Inhibition in Glioblastoma: Which Clinical Perspectives?

**DOI:** 10.3389/fonc.2021.772052

**Published:** 2021-11-03

**Authors:** Victoria Bouchè, Giovanni Aldegheri, Carmine Antonio Donofrio, Antonio Fioravanti, Samuel Roberts-Thomson, Stephen B. Fox, Francesco Schettini, Daniele Generali

**Affiliations:** ^1^ Department of Medicine, Surgery and Health Sciences, Cattinara Hospital, University of Trieste, Trieste, Italy; ^2^ Department of Neurosurgery, Manchester Centre for Clinical Neurosciences, Salford Royal National Health System (NHS) Foundation Trust, Manchester Academic Health Sciences Centre, University of Manchester, Manchester, United Kingdom; ^3^ Medical Oncology and Translational Research Unit, Azienda Socio-Sanitaria Territoriale (ASST) of Cremona, Cremona Hospital, Cremona, Italy; ^4^ Department of Pathology, Royal Melbourne Hospital, Parkville, VIC, Australia; ^5^ Department of Pathology, Peter MacCallum Cancer Centre, The University of Melbourne, Melbourne, VIC, Australia; ^6^ Translational Genomics and Targeted Therapies in Solid Tumors Group, August Pi I Sunyer Biomedical Research Institute (IDIBAPS), Barcelona, Spain; ^7^ Department of Medical Oncology, Hospital Clinic of Barcelona, Barcelona, Spain; ^8^ Unit of Neurosurgery, Azienda Socio-Sanitaria Territoriale (ASST) of Cremona, Cremona Hospital, Cremona, Italy

**Keywords:** glioblastoma, BRAF V600E, targeted therapy, BRAF-inhibitors, MEK-inhibitors, IDH, epithelioid glioblastoma

## Abstract

IDH-wild type (wt) glioblastoma (GB) accounts for approximately 90% of all GB and has a poor outcome. Surgery and adjuvant therapy with temozolomide and radiotherapy is the main therapeutic approach. Unfortunately, after relapse and progression, which occurs in most cases, there are very limited therapeutic options available. *BRAF* which plays a role in the oncogenesis of several malignant tumors, is also involved in a small proportion of *IDH*-wt GB. Previous successes with anti-B-Raf targeted therapy in tumors with V600E *BRAF* mutation like melanoma, combined with the poor prognosis and paucity of therapeutic options for GB patients is leading to a growing interest in the potential efficacy of this approach. This review is thus focused on dissecting the state of the art and future perspectives on *BRAF* pathway inhibition in *IDH*-wt GB. Overall, clinical efficacy is mostly described within case reports and umbrella trials, with promising but still insufficient results to draw more definitive conclusions. Further studies are needed to better define the molecular and phenotypic features that predict for a favorable response to treatment. In addition, limitations of B-Raf-inhibitors, in monotherapy or in combination with other therapeutic partners, to penetrate the blood-brain barrier and the development of acquired resistance mechanisms responsible for tumor progression need to be addressed.

## Introduction

Glioblastoma (GB) is the most frequent primary central nervous system (CNS) tumor, characterized by its aggressiveness and poor prognosis, with a median overall survival of only 14-20 months (1). Despite increasing knowledge of GB biology, the standard of care is still represented by radical surgical resection, if feasible, followed by adjuvant radiotherapy (RT) and chemotherapy (CT) with temozolomide (TMZ) (2). Unfortunately, relapse and progression occur in most cases and average overall survival (OS) is of no more than 5-7 months after relapse, due to the very limited therapeutic options available (2, 3).

The raf murine sarcoma viral oncogene homolog B (*BRAF*) proto-oncogene is located in the long arm of chromosome 7 (7q34) and encodes for the B-Raf protein, a serine/threonine kinase belonging to the RAF protein family, which also includes A-Raf and C-Raf (4). Raf proteins participate in the MAP kinase (MAPK) signaling pathway, that regulates some of the main cellular functions, including proliferation, differentiation, cell motility and apoptosis (4, 5).

Under normal conditions, the MAPK pathway is activated by several distinct stimuli, including growth factors, cytokines, and ceramides and is downregulated by physiological negative feedbacks (6). In the most characterized MAPK signaling cascade, ligand-induced extracellular binding to cognate receptors (usually a tyrosine kinase receptor [RTK]), induce activation of the Ras GTPase protein that binds to a Raf protein family member, which, in turn, leads to homo- or hetero-dimerize and triggering of a phosphorylation cascade resulting in cell proliferation and survival (**Supplementary Figure 1**) **(** 4, 6).

However, aberrant activation of the MAPK pathway is implicated in the development of many tumors. The most frequent mechanism is represented by *BRAF* gene activating mutations, which are present in ~7% of human cancers, including melanoma (40%), colorectal adenocarcinoma (15%), non-small cell lung cancer (NSCLC) (4%) and GB (8%) (7–9),

Following impressive beneficial effects obtained in *BRAF*-mutant melanomas with B-Raf-inhibitors and their subsequent approval, and considering the lack of therapeutic options for resistant/relapsing GB, there has been a growing interest in using this therapeutic approach has been rising in the last few years. This review of the literature focuses on the biological mechanisms that link *BRAF* mutations with cancer, the role of such mutations in the context of GB, along with potential therapeutic opportunities, pitfalls and challenges.

## *BRAF* Mutations: A Brief Overview

*BRAF* mutations can be divided into three classes (10, 11). Class I mutations are related to codon 600, a segment of the *BRAF* activating domain, and lead to the encoding of a constitutively activated protein. The amino acid substitution of glutamic acid with valine at codon 600 (V600E) accounts for more than 90% of cases. The V600E mutation induces a stabilization of the B-Raf protein, resulting in its activation without the need for dimerization. A smaller percentage of mutations includes other aminoacidic substitutions of codon 600, i.e. V600M, V600K, V600R, V600D (9, 12).

Class II mutations induce the constitutive activation of B-Raf through a Ras-independent dimerization. They are mostly caused by the K601E, K601N, K601T and L597Q substitutions at the *BRAF* activation segment and the G464, G469A, G469V, G469R substitutions within the P-loop. This class also includes *BRAF* gene fusions, the most common of which is the *KIAA1549-BRAF* (11–13).

Class III mutations are characterized by a reduced or absent kinase activity that leads to an alteration of both Ras and B-Raf heterodimers and enhancement of C-Raf-mediated MAPK activation. The main class III mutations are: D954N, N581S, G466V, D594G, G466E, G596D (11–13).

## *BRAF* Mutations in GB

Class I mutations, especially the V600E are present in ~7% of all CNS tumors; the frequency of class II and III mutations in CNS tumors has not been sufficiently documented. BRAF mutations are more prevalent in specific histotypes, with up to 60% of pleomorphic anaplastic xanthoastrocytomas, 20-70% of gangliogliomas, up to 10% of pilocytic astrocytoma and approximately 8% of GB (15-20% in pediatric cases and 3% of adult cases) harboring mutations (9, 14). Indeed, *IDH*-wild type (wt) GB, which are considered as primary tumors, have a higher prevalence of the V600E mutation compared with *IDH*-mutant GB, that develop from astrocytomas and are considered as secondary GB (15, 16). The correlation with specific GB histotypes has also been evaluated. More in detail, a study analyzing the characteristics of mutated GB in pediatric patients showed that all V600E-positive tumors were primary GB with no mutation in *IDH1 (* 17). Additionally, 2/3 of these mutated tumors presented with the giant cells histotypic variant, showing a heterogeneous distribution of the mutation, mostly present in the giant cells (17). Nevertheless, another study in giant cell GB failed to identify the mutation (18). Conversely, the mutations were observed in 50-93% of the rare GB epithelioid variant (18, 19). In fact, several studies have suggested that epithelioid GB is related both histologically and molecularly to anaplastic pleomorphic xanthoastrocytoma, which is frequently enriched in V600E B-Raf mutations, as already mentioned (20). The epithelioid GB is a rare and molecularly heterogenous subtype that, differently from classic GB, usually occurs in pediatric and young adult patients and is characterized by large epithelioid cells which vaguely remind of melanoma cells (**Supplementary Figure 2**). This histotype is also associated with a higher frequency of hemorrhage and leptomeningeal dissemination (21, 22). Nonetheless, no differences in the qualitative and quantitative histological features or any radiologic imaging features between mutated and wild-type epithelioid GB have been identified (18). Importantly, the presence of V600E mutation seemed to be associated with a relatively favorable prognosis (18). Yet, a cohort of epithelioid GB in older patients with frequent *BRAF* mutation and a molecular signature similar to IDH-wt GB has been characterized by having a highly aggressive behavior (20).

## B-Raf-Inhibitors in GB: Clinical Evidences

The B-Raf-inhibitors vemurafenib, dabrafenib and encorafenib entered the Oncology clinical practice few years ago, after demonstrating significant and profound survival improvements in V600E-mutant melanoma (23, 24). These molecules pertain to the second generation of selective B-Raf-inhibitors. These orally available, small molecules are effective on constitutively active mutant B-Raf monomers. Their main mechanism of action is based on the competitive occupation of the ATP binding pocket that stabilizes the Raf kinase in its active conformation, so to force the protein to take an inactive one (25). This results in the disruption of the downstream MAPK signaling pathway, leading to G1 cell-cycle arrest and apoptosis (25). Vemurafenib shows activity against V600E, V600D and V600R *BRAF* mutations and dabrafenib against the V600E, V600D, V600R and V600K, but both drugs are not capable of inhibiting wild-type *BRAF* or non-V600 mutations. Encorafenib is not only capable of targeting V600E and V600K *BRAF* mutantions, but also displays some inhibitory effect on wild-type *BRAF (* 25).

Although the rates of *BRAF* mutation in GB are far lower than in melanoma (8% *vs* 40%, approximately), in light of the limited therapeutic options available for refractory/relapsing GB and following the success of these drugs in other settings, there is a growing interest in establishing a potential role for B-Raf-inhibitors in this scenario.

To date, studies have shown promising results with second-generation B-Raf-inhibitors essentially in V600E-mutant CNS tumors, although data for the rarer variants (V600D, V600K, V600R) are still sparse.

Sporadic cases of impressive treatment responses with vemurafenib have been reported, including cases of complete radiological regression of the disease, with prolonged symptomatic control (26–28).

Remarkable therapeutic effects have also been documented with dabrafenib, resulting in disease stabilization for several months or years (29–31). Several studies in patients with brain metastases from *BRAF*-mutant melanoma showed higher response rates with dabrafenib compared to vemurafenib, suggesting that the former might better penetrate the blood-brain barrier (BBB) with respect to the latter, thanks to its smaller size (13).

Higher-quality evidence on the therapeutic efficacy of B-Raf-inhibitors in GB recently came from the VE-BASKET trial, a phase 2 study of non-melanoma tumors harboring *BRAF* V600E mutation (32). Patients were divided into seven cohorts. The first six included patients with prespecified tumors (NSCLC, ovarian, colorectal, and breast cancers, cholangiocarcinoma, and multiple myeloma), while the seventh cohort included other *BRAF* V600-mutant cancers. Among them, 24 patients with different mutant gliomas of any grade (6 properly affected by GB), received vemurafenib after tumor progression with standard therapy (32, 33). Patients >16 years old who had not received previous treatment with B-Raf or MEK-inhibitors were recruited, including some requiring minimal dosing of vemurafenib (480 mg twice daily) because of poor tolerance. Patients continued vemurafenib until tumor progression or intolerable toxicity. Key end-points included confirmed objective response rate (ORR) by RECIST version 1.1, progression-free survival (PFS), OS, and safety (32). Malignant diffuse gliomas (GB and anaplastic astrocytomas, 11 patients in total) showed an ORR of 9.1% (95% confidence interval [CI]: 0.2 – 41.3%) and a confirmed clinical benefit rate (CBR) of 27.3% (95%CI: 6.0 – 61.0%). Median PFS was 5.3 months (95% CI: 1.8 - 12.9 months), with a median OS of 11.9 months (95% CI: 8.3 - 40.1 months) (32). However, results were extremely heterogeneous depending on gliomas grade/histotypes. In fact, low-grade *IDH*-wt gliomas provided the most encouraging data, with the highest ORR, CBR, PFS and OS (32). Unfortunately, the trial did not provide an extensive molecular characterization of these tumors, nor patients were tested for the V600 *BRAF* mutations immediately before entering the trial. Therefore, possible reasons explaining these differential results might be either that some tumors might have been the result of the evolution of subclones with no *BRAF* alterations, or the intratumoral mutational heterogeneity, with *BRAF* being only one of multiple mutations that promoted tumor mass formation and growth (32). A phase I/II clinical trial also showed promising activity for dabrafenib in V600-mutant high-grade and low-grade gliomas (34). However, detailed results regarding the 2 included GB patients were not reported.

Current clinical data on pediatric/adult *BRAF*-mutant GB treated with B-Raf-inhibitors (alone or combined with other treatments) are provided in **Table 1**.

**Table 1:** Available clinical data on pediatric/adult *BRAF*-mutant glioblastoma treated with B-Raf-inhibitors.

First Author	Journal and Year	Study Type	CNS Tumor Type	N. Patients	Drug	Best Response	Treatment Duration/PFS	Final Outcome/OS
Burger MC	Oncology Reports 2017	Case Report	V600E GB	1	dabrafenib	Nearly CR	3 months	On treatment
Ceccon G	Int J Mol Sci 2018	Case Report	V600E eGB	1	dabrafenib	SD	10 months	PD
Qin C	Front Oncol 2020	Case Report	V600E GB	1	vemurafenib	PR	>2 years	On treatment
Robinson GW	BMC Cancer 2014	Case Report	V600E GB	1	vemurafenib	CR	6 months	On treatment
Beba Abadal K	JCO Precision Oncology 2017	Case Report	V600E GB	1	vemurafenib	PR	11 months	On treatment
Meletath SK	JNCCN 2016	Case Report	V600E HGG (GB/AGG)	1	dabrafenib + TTFields	CR	>2 years	On treatment
Schreck KC	JNCCN 2018	Case Report	V600E eGB	1	dabrafenib + trametinib	SD	16 months	On treatment
Kushnirsky M	JCO Precision Oncology 2020	Case Report	V600E GB	1	dabrafenib + trametinib	CR	11 months	On treatment
Kanemaru Y	Acta Neuropathol Commun 2019	Case Report	V600E eGB	1	dabrafenib + trametinib	PR	4 weeks	PD (after treatment suspension for lack of funding)
Johanns TM	J Natl Compr Canc Netw 2018	Case Report	Mixed V600E PXA/eGB	1	dabrafenib + trametinib + bevacizumab	PR	Approx. 4 months	PD
			V600E eGB	1	dabrafenib + trametinib	PR	11 months	PD
Kaley T	J Clin Oncol 2018	Basket Trial	V600E Malignant Diffuse Glioma	11*	vemurafenib	ORR 9.1% (95%CI: 0.2% - 41.3%); CBR 27.3% (95%CI: 6.0% - 61.0%)	mPFS 5.3 months (95% CI: 1.8 - 12.9)	mOS 11.9 months (95% CI: 8.3 - 40.1)
Kieran MW	Clin Can Res 2019	Phase I	V600 HGG and LGG	10°	dabrafenib	–	71.7 (5.7–130.4) weeks^#^	PD

CR, complete response; PR, partial response; SD, stable disease; PD, progressive disease; CI, confidence interval; mPFS, median progression-free survival; mOS, median overall survival; ORR, overall response rates; CBR, clinical benefit rate; eGB, epithelioid glioblastoma; HGG, high-grade glioma; LGG, low-grade glioma; PXA, anaplastic pleomorphic xanthoastrocytoma; AGG, anaplastic ganglioglioma; GB, glioblastoma; TTFields, Tumor-treating electromagnetic fields. *6 glioblastoma and 5 anaplastic astrocytoma; °2 glioblastomas; ^#^HGG + LGG.

## B-Raf Inhibition Pitfalls, Mechanisms of Resistance and Overcoming Strategies

Second-generation B-Raf-inhibitors present some limitations. Firstly, they can effectively inhibit B-Raf monomers but not dimers, which make them ineffective in case of class III mutations. Secondly, as observed in melanoma, the efficacy of the monotherapy with B-Raf-inhibitors is ultimately impaired by the development of acquired (secondary) resistance, usually consisting in the reactivation of MAPK by alternative pathways (35). It is estimated that more than 90% of patients with *BRAF* mutated melanoma develop resistance within 1 year of monotherapy. Several trials demonstrated that an effective way of overcoming this issue, was to combine B-Raf-inhibitors with MEK-inhibitors. This drug class (binimetinib, cobimetinib, trametinib) is able to prevent the activation of the protein ERK by inhibiting the downstream effector of B-Raf. When used alone they have not been found to be sufficiently effective, however in combination with B-Raf-inhibitors rapidly became the novel standard of care, replacing anti-B-Raf monotherapy, following significant improvements in ORR, PFS and OS obtained in phase III pivotal trials (36–38). A recent phase II study in *BRAF*-mutant NSCLC also showed positive ORR and survival outcomes, leading to the approval of such combinations also in this context (39). Currently US Food and Drug Administration (FDA)-approved combinations are vemurafenib+cobimetinib in melanoma, encorafenib+binimetinib in melanoma, dabrafenib+trametinib in melanoma and NSCLC. There is emerging evidence supporting the hypothesis of a synergic effect of this combination therapy also in CNS tumors, which are not exempt from the development of resistance (40).

A case report from Kushnirsky et al. demonstrated prolonged complete response with the combination of dabrafenib and trametinib after B-Raf inhibition failure in *BRAF*-mutant GB (40). Another study reporting the clinical history of a patient with an *IDH*-wt epithelioid GB, showed an impressive disease regression with dabrafenib+trametinib therapy administered after the development of spinal metastases, a severe condition usually associated with a 3-month prognosis (21). Other reports also showed rapid and clinically meaningfully responses to dabrafenib+trametinib combinations, though highlighting the development of a rapidly evolving disease after the insurgence of resistance (41, 42).

There are currently many active phase I and/or II clinical trials evaluating the tolerability, activity and efficacy of targeted therapies with B-Raf-inhibitors, with or without MEK-inhibitors, in patients with *IDH*-wt gliomas of both low and high grade, whose results will be important for a more comprehensive and clearer overview of the real potential of this therapeutic strategy in the next future (**Table 2**).

**Table 2:** Current clinical trials of B-Raf and/or MEK inhibitors in different grade BRAF-mutated gliomas.

STUDY PHASE AND PURPOSE	RECRUITMENT STATUS	TREATMENT	DISEASE
***PHASE I STUDY***	Active, not recruiting	Vemurafenib	▪ Pediatric Recurrent/Refractory BRAFV600E-mutant Gliomas
Vemurafenib in Children With Recurrent/Refractory BRAF Gene V600E (BRAFV600E)-Mutant Gliomas			
***PHASE I STUDY***	Recruiting	ABM-1310 Cobimetinib	▪ Advanced Solid Tumor ▪ BRAF V600 Mutation
Safety of ABM-1310 in Patients With Advanced Solid Tumors			
***PHA SE I, II STUDY***	Recruiting	Dabrafenib	▪ Low Grade Glioma (LGG) of Brain With BRAF Aberration ▪ High Grade Glioma (HGG) of the Brain With BRAF Aberration ▪ Low Grade Glioma of Brain With Neurofibromatosis Type 1
A Trial of Dabrafenib, Trametinib and Hydroxychloroquine for Patients With Recurrent LGG or HGG With a BRAF Aberration		Trametinib	
		Hydroxychloroquine	
***PHASE I, II STUDY***	Completed	Trametinib	▪ Cancer
Study to Investigate Safety, Pharmacokinetic (PK), Pharmacodynamic (PD) and Clinical Activity of Trametinib in Subjects With Cancer or Plexiform Neurofibromas and Trametinib in Combination With Dabrafenib in Subjects With Cancers Harboring V600 Mutations		Dabrafenib	
***PHASE I, II STUDY***	Active, not recruiting	Selumetinib	▪ Low Grade Glioma ▪ Recurrent Childhood Pilocytic Astrocytoma ▪ Recurrent Neurofibromatosis Type 1 ▪ Recurrent Visual Pathway Glioma ▪ Refractory Neurofibromatosis Type 1 ▪ Refractory Visual Pathway Glioma
Selumetinib in Treating Young Patients With Recurrent or Refractory Low Grade Glioma			
***PHASE II STUDY***	Recruiting	Encorafenib	▪ High Grade Glioma ▪ BRAF V600E ▪ BRAF V600K ▪ Anaplastic Astrocytoma ▪ Anaplastic Pleomorphic Xanthoastrocytoma ▪ Gliosarcoma ▪ Glioblastoma
Study of Binimetinib With Encorafenib in Adults With Recurrent BRAF V600-Mutated HGG (BRAF)		Binimetinib	
***PHASE II STUDY***	Recruiting	Dabrafenib Mesylate	▪ Anaplastic Astrocytoma ▪ Anaplastic Ganglioglioma ▪ Anaplastic Pleomorphic Xanthoastrocytoma ▪ Glioblastoma ▪ Malignant Glioma ▪ WHO Grade III Glioma
Dabrafenib Combined With Trametinib After Radiation Therapy in Treating Patients With Newly-Diagnosed High-Grade Glioma		Radiation Therapy	
		Trametinib Dimethyl Sulfoxide	
***PHASE II STUDY***	Recruiting	Trametinib	▪ Low-grade Glioma ▪ Plexiform Neurofibroma ▪ Central Nervous System Glioma
Trametinib for Pediatric Neuro-oncology Patients With Refractory Tumor and Activation of the MAPK/ERK Pathway			
***PHASE II STUDY***	Recruiting	Vemurafenib	▪ Advanced Malignant Solid Neoplasm ▪ Ann Arbor Stage III Childhood Non-Hodgkin Lymphoma ▪ Ann Arbor Stage IV Childhood Non-Hodgkin Lymphoma ▪ Ependymoma ▪ Ewing Sarcoma ▪ Hepatoblastoma ▪ Langerhans Cell Histiocytosis ▪ Malignant Germ Cell Tumor ▪ Malignant Glioma ▪ Osteosarcoma ▪ Peripheral Primitive Neuroectodermal Tumor ▪ Recurrent Childhood Central Nervous System Neoplasm ▪ Recurrent Childhood Non-Hodgkin Lymphoma ▪ Recurrent Malignant Solid Neoplasm ▪ Recurrent Neuroblastoma ▪ Refractory Malignant Solid Neoplasm ▪ Refractory Neuroblastoma ▪ Refractory Non-Hodgkin Lymphoma ▪ Refractory Primary Central Nervous System Neoplasm ▪ Rhabdoid Tumor ▪ Rhabdomyosarcoma ▪ Soft Tissue Sarcoma ▪ Wilms Tumor
Vemurafenib in Treating Patients With Relapsed or Refractory Advanced Solid Tumors, Non-Hodgkin Lymphoma, or Histiocytic Disorders With BRAF V600 Mutations (A Pediatric MATCH Treatment Trial)			
***PHASE II STUDY***	Active, not recruiting	Dabrafenib Trametinib Carboplatin with vincristine	▪ Diffuse Astrocytoma ▪ Anaplastic Astrocytoma ▪ Astrocytoma ▪ Oligodendroglioma, Childhood ▪ Anaplastic Oligodendroglioma ▪ Glioblastoma ▪ Pilocytic Astrocytoma ▪ Giant Cell Astrocytoma ▪ Pleomorphic Xanthoastrocytoma ▪ Anaplastic Pleomorphic Xanthoastrocytoma ▪ Angiocentric Glioma ▪ Chordoid Glioma of Third Ventricle ▪ Gangliocytoma ▪ Ganglioglioma ▪ Anaplastic Ganglioglioma ▪ Dysplastic Gangliocytoma of Cerebrellum ▪ Desmoplastic Infantile Astrocytoma and Ganglioglioma ▪ Papillary Glioneuronal Tumor ▪ Rosette-forming Glioneurona Tumor ▪ Central Neurocytoma ▪ Extraventricular Neurocytoma ▪ Cerebellar Iponeurocytoma
Phase II Pediatric Study With Dabrafenib in Combination With Trametinib in Patients With HGG and LGG			
***PHASE IV STUDY***	Recruiting	Dabrafenib	▪ Melanoma ▪ Non-Small Cell Lung Cancer ▪ Solid Tumor ▪ Rare Cancers ▪ High Grade Glioma
Dabrafenib and/or Trametinib Rollover Study		Trametinib	

HGG, high-grade gliomas; LGG, low-grade gliomas.

Some of the most characterized mechanisms of resistance to second-generation B-Raf-inhibitors include Raf isoform switching, activation of RTKs, like IGF-1R or EGFR, and the activation of the PI3K pathway (e.g. through PTEN loss or activating mutation in PIK3CA) to promote cell survival (35). Still, many mechanisms of resistance have yet to be fully understood. Most of this knowledge is currently coming from different tumor settings.

With respect to isoform switching, B-Raf inhibition that blocks the phosphorylation of ERK also inhibits the negative feedback control on the Ras/Raf/MAPK pathway and may result in a reactivation of MAPK through C-Raf-mediated dimerization. Second-generation B-Raf-inhibitors are not effective in this case (35, 43). To overcome this issue, several molecules are currently under different stages of development in largely non-CNS context and include: 1) dual B-Raf and Src inhibition; 2) selective disruption of the B-Raf-mediated signaling in mutant cells without affecting normal MAPK pathway activation, so to reduce on-target toxicities and development of second tumors (so-called paradox breakers); 3) binding to the DFG-OUT (inactive) conformation of the B-Raf kinase; 4) pan-Raf inhibition; 5) inhibition of both Raf’s dimeric and monomeric forms, so to potentially overcome the resistance resulting from Raf dimerization (43).

Another potential cause of resistance to B-Raf-inhibitors is the previously mentioned reactivation of the MAPK pathways *via* hyperactivation of RTK. In this perspective, the amplification of EGFR has been observed as a common alteration in GB that can reactivate MAPK through alternative mediators such as C-Raf, resulting in the formation of C-Raf/B-Raf heterodimers. Notably, this mechanism of resistance can affect not only the B-Raf monotherapy but also anti-B-Raf and MEK combinations, as observed in patients with mutant EGFR (35, 43). These mutational profiles have prompted the need to evaluate triple combination therapies: currently, triple therapy with dabrafenib+trametinib+panitumumab (anti-EGFR) is being evaluated in colorectal cancer with EGFR mutation (35, 43).

As above, mutations in the PI3K/AKT pathway can cause the reactivation of MAPK. As a consequence, a potential strategy to overcome this form of acquired resistance is to use a therapeutic regimen including a PI3K-inhibitor. In this perspective, a phase Ib/II study with encorafenib+cetuximab (an EGFR-inhibitor) with or without alpelisib (a PI3K-inhibitor) in *BRAF*-mutant colorectal cancer was conducted. It showed promising clinical activity with increased toxicity but also a slight PFS benefit related to the addition of alpelisib, opening up to the possibility of further exploring this therapeutic strategy in other *BRAF*-mutant tumors (44).

In addition to the problem of acquired resistance, some intrinsic constitutive characteristics of *IDH-*wt GB might be responsible for *de novo* (primary) resistance, which might explain the conflicting results observed up to now. In this perspective a relevant issue is represented by the frequent intratumoral histological and molecular heterogeneity of GB (18, 19, 42, 45, 46). Mixed histologies or differential distribution of B-Raf mutation within different tumor areas may lead only to partial regressions, without counteracting the entire tumor growth, potentially determining also a selection of resistant areas (18, 19, 42, 45, 46).

Another important treatment limitation is represented by the reduced capabilities of many drugs to cross the BBB. In this regard we can extrapolate evidences from studies that evaluated this issue in melanoma brain metastases. An interesting study carried out by Sakji-Dupré et al. demonstrated not only that vemurafenib has a limited ability to cross the BBB but also that concentrations in the cerebro-spinal fluid (CSF) and plasma are not strictly correlated. This leads to the hypothesis that there are several interindividual factors that might influence the BBB permeability to BRAF inhibitors (47). For example, 99% of B-Raf-inhibitors bind to albumin in the bloodstream (48, 49), with only the remaining free unbound portion being able to cross the BBB. Consequently, the proportion of an administered inhibitor effectively passing the BBB might vary depending on albumin levels from patient to patient. In this context, hypoalbuminemia might determine higher concentration of B-Raf-inhibitors in the CNS, as well as hyperalbuminemia might cause the opposite effect. Nevertheless, there are no studies currently supporting this hypothesis, nor its potential therapeutic implications and it should be considered as highly speculative.

The integrity of the BBB is another crucial factor influencing drug capability of penetrating into the CNS. Following this assumption, Narayana et al. combined B-Raf-inhibitors to brain RT so to test if temporary destruction of the BBB induced by RT in melanoma patients with brain metastases might favor CNS penetration of anti-B-Raf (50). Although results seem to support this hypothesis, further studies are needed, especially in the context of primary CNS tumors.

Another determinant factor seems to be the action of two proteins that work as efflux transporters and actively extrude vemurafenib out of the brain compartment, namely the glycoprotein P (Pgp) and the breast cancer resistance protein (BCRP). Durmus et al. administered to mice the Pgp and BCRP inhibitor elacridar, in combination to vemurafenib to observe if this strategy might improve the penetration of the latter in the CNS. After the administration of elacridar, vemurafenib concentration increased by 2.5 times in serum and by 9.4 times in the CSF (51). The therapeutic implications of this preclinical study have to be further explored in clinical trials.

Finally, different targeted drugs might have distinct BBB penetration capabilities, rather limited in the case of anti-MEK drugs (52). This might concur to impair the therapeutic efficacy of B-Raf-inhibitors+MEK-inhibitors combination. For this reason, the development of brain penetrant B-Raf- and MEK-inhibitors is currently underway (13, 53). One of the most promising molecule is the potent brain-penetrant B-Raf inhibitor PF-07284890, whose pharmacokinetics, safety and efficacy are under evaluation in a first-in-human trial, in combination or not with binimetinib, in patients with V600-mutant solid tumors (54).

## Discussion

Overall, B-Raf mutation occurs mostly in the rare epithelioid variant of IDH-wt GB and hence appear to be more frequent in GB of patients <30 years old. Evidence mostly coming from case reports or experiences in other solid tumors (especially melanoma and NSCLC) suggest that B-Raf-inhibitors, potentially in combination with MEK-inhibitors, might be a valuable therapeutic option for this rare cancer. In any case, the mainstay of treatment remains surgery followed by RT and TMZ, which, at present, provides better outcomes than B-Raf inhibition, which should be pursued only in a refractory/relapsing setting, or in case of ineligibility for standard therapy. In this perspective, the screening of GB patients for the detection of V600 *BRAF* mutations, especially when under 30 years of age, should be undertaken. Results from ongoing clinical trials are also of utmost importance to draw definitive conclusions regarding whether, when and to whom administer B-Raf-inhibitors, combined or not with MEK-inhibitors.

In conclusion, although the evidence at our disposal are still quantitatively few and qualitatively scarce, the potential for B-Raf inhibition in the context of *BRAF-*mutant GB merit further investigation, since it might represent a valuable therapeutic opportunity for at least a proportion of patients affected by this lethal disease.

## Author Contributions

VB, GA, CD, AF, FS, and DG conceived the study. VB and FS performed the literature search. VB, DG, and FS wrote the first manuscript draft. SR-T provided part of the images for **Supplementary Figure 2**. All authors revised the manuscript and approved the final submitted version.

## Funding

This study was supported by Mednote, spin-off - University of Trieste, within the Mozart Program.

## Conflict of Interest

The authors declare that the research was conducted in the absence of any commercial or financial relationships that could be construed as a potential conflict of interest.

## Publisher’s Note

All claims expressed in this article are solely those of the authors and do not necessarily represent those of their affiliated organizations, or those of the publisher, the editors and the reviewers. Any product that may be evaluated in this article, or claim that may be made by its manufacturer, is not guaranteed or endorsed by the publisher.
