# Supplementary material for: BRAF Signaling Inhibition in Glioblastoma: Which Clinical Perspectives?
Source: Front Oncol. 2021 Nov 3;11:772052. doi: 10.3389/fonc.2021.772052 (PMC8595319; doi:10.3389/fonc.2021.772052)
Supplement: Supplementary file 1 [file DataSheet_1.docx]

**Supplementary materials**

**Supplementary figure 1. The Ras-Raf-MAP kinase cascade**

**Legend.** The Ras-Raf-MAP kinase (MAPK) cascade is represented along with main B-Raf and MEK-inhibitors. The Ras GTPase causes the dimerization of Raf. After dimerization, Raf phosphorylates and activates MEK1/2, which, in turns, phosphorylate and activate the MAPK ERK1/2, ultimately resulting in the activation of transcription factors involved in cell survival, differentiation and proliferation, like ELK1, MYC or ETS1/2(3). RTK: Tyrosin Kinase Receptor; P: phosphorylated.

**Supplementary figure 2. Representative images of epithelioid and giant cell glioblastoma stained with H&E.**

**Legend. A:** IDH1 wild type (IHC negative) epithelioid GB showing a highly cellular tumor composed of epithelioid cells with marked nuclear pleomorphism, vesicular chromatin, prominent nucleoli, abundant eosinophilic cytoplasm and a prominent vasculature. **B:** Giant cell GB containing numerous large cells that demonstrate marked nuclear pleomorphism and atypia. The nuclei are large, frequently multinucleate with some bizarre forms. **C:** Epithelioid GB with BRAF-mutant protein detected via BRAF VE1 antibody with light hematoxylin counterstain. IHC: immunohistochemistry; H&E: hematoxylin and eosin; GB: glioblastoma.
